# Carbon Nanostructures Doped with Transition Metals for Pollutant Gas Adsorption Systems

**DOI:** 10.3390/molecules26175346

**Published:** 2021-09-02

**Authors:** J. M. Ramirez-de-Arellano, M. Canales, L. F. Magaña

**Affiliations:** 1 Escuela de Ingeniería y Ciencias, Tecnologico de Monterrey, Av. Eugenio Garza Sada 2501, Monterrey 64849, Mexico; jramirezdearellano@tec.mx; 2 Universidad Autónoma Metropolitana Unidad Azcapotzalco, Av. San Pablo Xalpa No. 180, Colonia Reynosa Tamaulipas, Delegación Azcapotzalco, Ciudad de México 02200, Mexico; monic.canales@gmail.com; 3 Instituto de Física, Universidad Nacional Autónoma de Mexico, Apartado Postal 20-364, Ciudad de México 01000, Mexico

**Keywords:** carbon nanostructures, doping, adsorption, transition metals, pollutant gas, nanotubes, graphene, fullerenes, graphdiyne, buckybowls

## Abstract

The adsorption of molecules usually increases capacity and/or strength with the doping of surfaces with transition metals; furthermore, carbon nanostructures, i.e., graphene, carbon nanotubes, fullerenes, graphdiyne, etc., have a large specific area for gas adsorption. This review focuses on the reports (experimental or theoretical) of systems using these structures decorated with transition metals for mainly pollutant molecules’ adsorption. Furthermore, we aim to present the expanding application of nanomaterials on environmental problems, mainly over the last 10 years. We found a wide range of pollutant molecules investigated for adsorption in carbon nanostructures, including greenhouse gases, anticancer drugs, and chemical warfare agents, among many more.

## 1. Introduction

**Figure 1:**
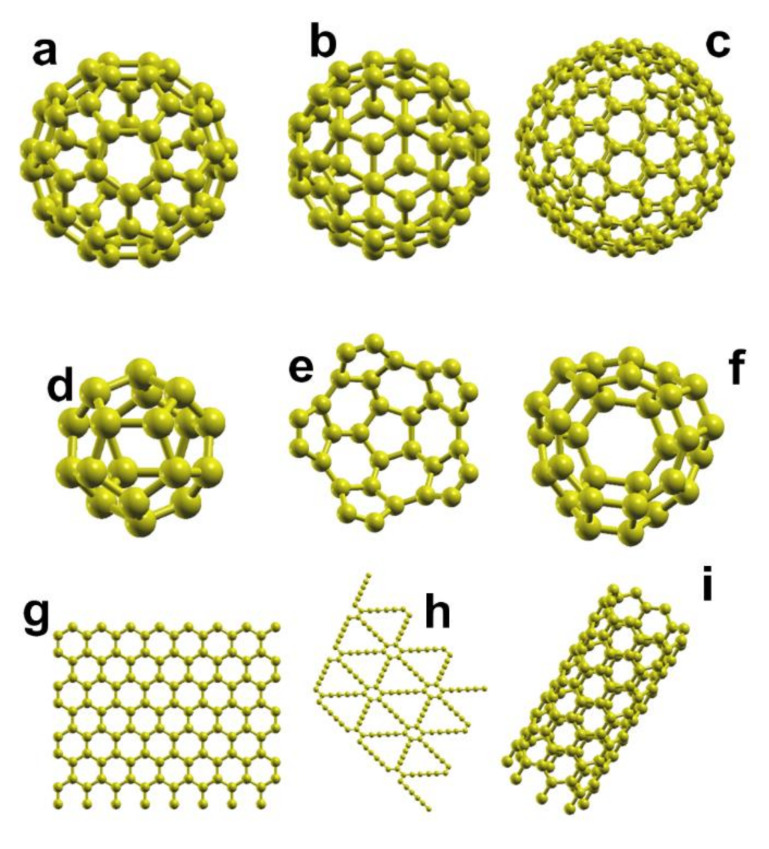
These are some of the carbon nanostructures reported experimentally. In (**a**,**b**), we have C_60_; we present the C_180_ in (**c**); C_20_ in (**d**); the buckybowl C_30_ in (**e**,**f**); graphene in (**g**); graphdiyne in (**h**); a single-walled carbon nanotube (SWCNT) in (**i**).

**Figure 2:**
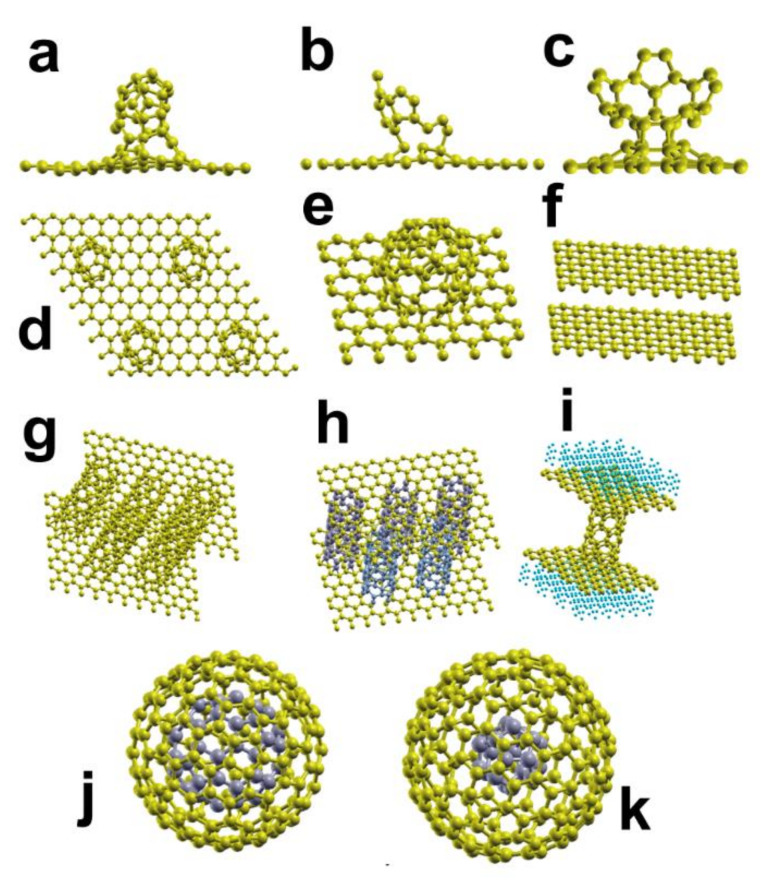
We present some of the hybrid carbon nanostructures reported experimentally. In (**a**–**d**), we have a C_30_ (a buckybowl) adsorbed on graphene; in (**e**), we show a fullerene C_60_, adsorbed on graphene; we show in (**f**), a bi-layer graphene; in (**g**,**h**), we present pillared graphene with nanotubes (SWCNTs); in (**i**) we show pillared graphene with hydrogen; we present in (**j**,**k**), two examples of carbon nano onions (CNO).

Since their discovery in 1985, the study of fullerenes and their exciting properties led to the subsequent discovery of many other carbon nanoforms: nanotubes, carbon onions, graphene, graphdiyne, carbon nanotori, etc., including hybrid nanostructures [1,2,3,4,5,6], see Figure 1 and Figure 2. As a result, there are numerous practical applications of carbon nanostructures, like gas sensors, adsorption of pollutant molecules, electric batteries, hydrogen adsorption, electronic devices, etc.

The capture of gases like CO, CO_2_, N_2_O, CH_4_, and many others is urgent. CO comes mainly from the non-complete combustion of fuels that contain carbon, and it is toxic and an asphyxiant. On the other hand, plenty of industrial processes in our world generate greenhouse gases like CO_2_, N_2_O, and CH_4_. Thus, humankind’s climate change and ecological challenges have increased the interest in carbon nanostructures for fast green applications [7].

We should mention that the specific surface area of graphene is 2630 m^2^/g, more significant than that of zeolites (between 500 and 800 m^2^/g) although smaller than that of metal-organic frameworks (or MOFs), which is around 7000 m^2^/g.

Thus, carbon nanostructures’ sizeable specific surface area is an excellent appeal for gas sensing and gas adsorption systems. Furthermore, the adequate doping of these structures may increase their adsorption capability and tune their selective adsorption. In particular, the reactivity of transition metals makes them ideal dopant candidates. Investigations based on the density functional theory (DFT) have been crucial to explore the adsorption properties of the different carbon nanostructures. Some researchers studied the variability in results obtained from diverse DFT implementations. For instance, predictions made using the Augmented Plane Waves plus local orbitals method (APW+lo) and the Projector Augmented Wave method (PAW) are identical for practical purposes [8]. Most of the computational studies reviewed in this paper also report the density of states (DOS) spectrum to complement their investigations on carbon nanostructures. They also include Mulliken or Lowdin charge analysis, electron density differences, HOMO and LUMO analysis, etc. In general, DFT is very efficient in computing interaction energies, while the Grand Canonical Monte Carlo (GCMC) calculations are adequate to predict adsorption capacities.

This work aims to review the most important and recent developments related to the adsorption of pollutants or hazardous gases by using carbon nanostructures. Among the most investigated pollutant gases, we must mention carbon monoxide (CO), carbon dioxide (CO_2_), methanol (CH_3_OH), methane (CH_4_), nitrogen monoxide (NO), nitrogen dioxide (NO_2_), ozone (O_3_), and formaldehyde (CH_2_O) with many others. However, we found some works on hydrogen adsorption on carbon nanostructures, which is essential to serve as a non-pollutant fuel. Although, we should mention that the number of works on this subject in the last 10 years is considerably less than previously.

We covered a 10-year range approximately, not in an exhaustive way, but describing the state-of-the-art up to date. When results were considered relevant enough, or when we believed that they would add important information for the context of the topic covered, we also included works dating from earlier years.

In the second section, we review the adsorption of molecules on undoped or nonmetal functionalized carbon nanostructures. We considered the subsections of nanotubes, graphene, fullerenes, graphdiyne, and hybrid systems. The third section reviews the same carbon structures as in the second section but doped with transition metals. Our conclusions are in the fourth section.

## 2. Adsorption of Molecules on Pristine or Nonmetal Functionalized Systems

### 2.1. Nanotubes

Various experimental studies focus on the gas adsorption capabilities of single-walled carbon nanotubes (SWCNTs) and multiple walled carbon nanotubes (MWCNTs), showing a good agreement with the Langmuir adsorption model. The nature of the gas sensing mechanism in nanotubes would be due to charge transfer between them and the molecules adsorbed, or due to effects of the carbon nanotube network, for devices made of bundles [9].

Several works using first-principles calculations have also shed light on this topic. Numerical DFT simulations using the local density approximation (LDA) for the exchange-correlation potential show that most gas molecules adsorb weakly on pristine SWCNTs. Still, certain gases like NO_2_ and O_2_ are more sensitive to be adsorbed due to the electronic properties of the former [10]. Although LDA is usually inaccurate to describe long-range interactions, the molecule-tube interaction seems to be between the LDA and the generalized gradient approximation (GGA). Hydrogen adsorption is another broad field of interest due to its potential applications as a non-pollutant fuel. Experimental studies suggest that fold-structure adsorbents convey better H_2_ adsorption than pore or flat structures [11]. It is common to characterize the carbon material by N_2_ adsorption at a given temperature and correlate that to their hydrogen storage capacity. SWNTs perform below activated carbon samples for hydrogen uptake, but the process is reversible in both cases. The storage capacity of the latter is about 4.5 wt% at 77 K [12]. Functionalizing MWCNTs using acids (F-MWCNTs) or by KOH activation (A-MWCNTs) increases the H_2_ gas sensing response [13,14].

The adsorption uptake of CO_2_ in pristine SWCNTs is more significant than that of CO, CH_4_, N_2_, and H_2_, a result that is confirmed both experimentally and by Monte Carlo simulations [15]. Experimental results show that carbon nanotubes, when modified by 3-aminopropyl-triethoxysilane (APTS), adsorb CO_2_ better. This effect decreases with temperature and increases with water content in the air. MWCNTs modified by APTS have good adsorption performance at 20 °C. The interaction between molecules and CNTs-APTS implies physisorption and chemisorption that shows stability for a cyclic operation. This operation is enhanced using saturated water vapor in the gas stream, giving CO_2_ deconcentration of around 67% [16,17,18]. Carbon nanotubes films have also been studied as CO_2_ sensors, both experimentally and via grand canonical Monte Carlo (GCMC) simulations [19].

Some other ways of increasing the adsorption capacity of CO_2_ are by functionalizing carbon nanotubes with nitrogen groups (N-MWCNTs); by fabricating polyethylenimine-polyethylene glycol/MWCNTs bilayer-structure devices—obtaining CO_2_ desorption without heating; or by a two-step modification of MWNTs using a mixture of diluted nitric and sulfuric acid and then 1,3-diamino propane [20,21,22].

The diameter and chirality of CNTs affect the adsorption. Flexible SWCNTs would adsorb up to 35 wt% of CO_2_, as studied using molecular dynamics (MD) calculations, including ad hoc potentials built to model the intramolecular interactions within the SWCNT [23].

The selectivity of CO_2_/CH_4_—i.e., good adsorption of CO_2_ combined with the exclusion of CH_4_—also increases by this functionalization, an effect that reaches an optimal value at lower pressures and room temperature, which is essential for industrial gas separation. Oxygen-rich surface functional groups attached to the surface of MWCNTs also increase the CO_2_ adsorption capacity [20,24]. MWCNTs physisorb methane (CH_4_) and natural gas showing fast sorption kinetics, making MWCNTs a promising porous media for natural gas storage [25]. Applying high pressures to double-walled carbon nanotube arrays increases the CO_2_ and N_2_ gas adsorption, an effect that is also obtained by functionalizing the tubes via oxygen plasma treatment [26]. GCMC simulations also indicate that water molecules can increase the selectivity of CO_2_ when CO_2_/CH_4_ and H_2_O/CO_2_/CH_4_ mixtures are considered in CNTs and silicon carbide-derived carbon (SiC-DC) [27].

Sulfur hexafluoride (SF_6_) is another greenhouse gas, considered several orders of magnitude more nocive than CO_2_, measured over long periods. MWNTs modified via H_2_SO_4_/H_2_O_2_ oxidation, or KOH activation, are suitable SF_6_ adsorbents [28].

The adsorption of acetone (C_3_H_6_O) is another issue extensively studied. This substance is liquid at room temperature, and it has a low boiling point of 329.20 K or 56.05 °C [29]. SWCNTs can strongly adsorb acetone molecules and then desorb them with increasing temperature within the interval of 400–900 K. For large-diameter SWCNTs, adsorption can happen in the interstitial channels, the bundles. The elastic deformation of the tubes can also increase the adsorption of acetone molecules [30]. Similar studies have been performed combining experimental and DFT methods to study the ethanol (C_2_H_5_OH) sensing properties of CNTs [31]. Polyaniline functionalized multiwalled carbon nanotubes (PANI/MWCNTs) have also been experimentally shown to detect ammonia (NH_3_) gas [32].

There are other ways of increasing the sensing performance of CNTs, besides deformation. For instance, sorted semiconducting nanotubes are more sensitive to pollutant gases like NO_2_ and NH_3_ than unsorted tubes [33]. For nitric oxide (NO) adsorption, in situ ultraviolet (UV) light illumination of pristine carbon nanotubes can increase their performance. The cleaning of the surface of the tubes by UV light would improve their gas sensing capacity [34]. Chemical polymerization with pyrrole (PPy) to obtain SWNTs/PPy nanocomposites can also enhance their sensitivity 10 times [35].

We found investigations of recent years on the adsorption of other gas molecules, like Xe, natural gas, or the nitrate ion NO_3_. The adsorption of Xe by single-walled carbon nanotubes (SWCNTs) has been studied experimentally and using ab initio calculations. Closed SWCNTs can be opened by oxidation at their ends and wall defect sites using ozone. This opening enhances the adsorption of Xe, as the nanotube etching increases wall openings at an optimal radius of 5–7 Å. After these optimal values, the adsorption rate drops [36]. SWCNTs can also adsorb a nitrate ion NO_3_^−^ in its gas phase [37].

Carbon nanotubes can also adsorb formaldehyde (HCHO) molecules in their interior and exterior walls, with preferential adsorption on the latter. The adsorbed HCHO molecules alter the electronic structure of the CNNTs, reducing the HOMO/LUMO gap from its original value of 4.02 to 2.44 eV, according to ab initio calculations [38]. These tubes can adsorb mixtures of SO_2_/N_2_ at the equimolar ratio, with MD calculations showing an increase in SO_2_ adsorption as the CNT diameter increases [39]. Finally, experimental studies have demonstrated the feasibility of MWCNTs as adsorbents of several volatile organic compounds (VOCs) such as benzene, methanol, ethanol, acetone, etc. [40].

Table 1 summarizes the adsorption energies—for the studies that reported them—and the characteristics of investigations covered in this section. When the study considers different variations or adsorption conditions for the same adsorbate, an energy range is given instead of a particular value.

### 2.2. Graphene

Typical calculations of molecules adsorption on graphene consider either a 4 × 4 or a 5 × 5 unit cell to avoid spurious interactions, as many codes work with periodic boundary conditions. The three adsorption sites considered in these studies are (a) at the bond between two C atoms or a B-site; (b) at the top of a C atom or T-site; (c) over the center of a hollow hexagon of C atoms, or H-site. Most DFT calculations work with a graphene monolayer, but some studies consider two or more sheets.

The adsorption of atomic or molecular hydrogen on graphene has attracted much interest in the last decade. Graphene can adsorb H atoms with binding energies per atom of about 0.8–1.9 eV [42]. There are predictions of theoretical calculations that hydrogenated graphene sheet would be a semiconductor. Deuterium (D) has been incorporated into graphene via thermal annealing in a process above 400 °C, which is not entirely reversible [43] to verify those predictions. Defects in graphene can also improve H_2_ adsorption. Defective graphene V_222_ is obtained by removing a C atom from the graphene sheet and then di-hydrogenating the three C atoms on the vacancy edges. MD calculations using a V_222_ structure show an H_2_ adsorption–desorption process that is more reversible than pristine graphene [44].

It is frequent to find graphene used as a substrate to adsorb H_2_O, NH_3_, CO, NO_2_, and NO, as explored via DFT ab initio calculations and experimentally. It is well established that pristine graphene adsorbs H_2_O molecules, with the latter acting as an acceptor. NO_2_ can also be physisorbed on pristine graphene, and it can induce more significant doping than NO [45,46]. Pristine graphene can physysorb NO molecules with electrons transferred from the first to the second, with the B-site being the most stable adsorption site [47]. It can also weakly adsorb hydrogen sulfide (H_2_S) and methane (CH_4_) [48]. NonTM-functionalized graphene can also detect ammonia (NH_3_). A way to increase the sensing performance of graphene is to produce pristine graphene noncovalently functionalized, usually by a biocompatible stabilizer such as flavin mononucleotide sodium salt [49].

From experimental and theoretical investigations, we find that graphene can adsorb organic molecules such as acetone, acetonitrile, dichloromethane, ethanol, ethyl acetate, hexane, and toluene. Graphene modeled as coronene would interact with said molecules primarily by London dispersive forces [50]. Table 2 summarizes the main results reviewed in this section.

### 2.3. Fullerenes

Fullerenes can be obtained directly from graphene [61]. They are the subject of a great deal of experimental and theoretical research due to their exciting adsorption properties given by their characteristics. From first-principles studies we know that the size of the fullerene impacts its stability and its hydrogen storage capacity. The cohesive and formation energies of fullerenes decrease with increasing size [62].

Furthermore, fullerenes adsorb gases like N_2_, Ar, and CO_2_, and for the latter, an exceptionally large C_460_ fullerene would adsorb up to 28 wt% at room temperature [62]. A P-doped fullerene can adsorb the CO_2_ molecules; such doping increases the selectivity of CO_2_ concerning an N_2_/CO_2_ mixture, as shown by DFT studies. Additionally, an electric field applied to the systems affects the interaction between the P-doped-CO_2_ and the CO_2_ molecule, going from physisorption to chemisorption [63].

Monte Carlo simulations show that C_60_ fullerenes can also adsorb ethylene (C_2_H_4_) at 150 K [64]. Experimental results show that self-assembled C_60_ crystals have excellent sensing properties to detect toxic aromatic solvent vapors such as aniline, toluene, benzene, ethanol, hexane, cyclohexane, and methanol [65].

Additionally, DFT studies showed that fullerene-based devices reduced nitrous oxide (N_2_O) and carbon monoxide (CO) pollution. In that case, Si-coordinated nitrogen-doped C_60_ fullerenes, labeled as Si@C_54_N_4_, catalyzes the N_2_O reduction and CO oxidation in the presence of an O_2_ molecule [66]. In [67], the authors found that metal oxides (MOxs) such as Cu2O, ZnO, and NiO, when adsorbed on C_60_ fullerenes, can adsorb nitrogen dioxide (NO_2_) as well as CO much better than C_60_ alone. This is caused by a more significant charge transfer, more considerable adsorption energies, and more extensive enthalpy changes when compared to those of the pristine C_60_ fullerene.

Such pristine C_60_ fullerenes can adsorb H_2_, and their interaction has been studied by several means, including a modification of the L-J potential and the continuum approach [68]. When C_60_ fullerenes oxidize at 400 °C and a pressure of 2 bar, they enhance their surface and increase H_2_ adsorption. Experimental studies showed that this process increases the H_2_ adsorption by a factor of three, at 77 K and a pressure of 120 bar [69]. When C_60_ or C_70_ fullerenes are doping helium droplets, they can physisorb both hydrogen H_2_ and deuterium D_2_ [70]. Furthermore, in [62], the authors showed that the previously mentioned C_460_ fullerene would reach H_2_ storage of 7.60 wt% at 300 K.

There are theoretical investigations to compare the efficiency of the porous structure of fullerenes with that of similar hexagonal boron nitride (hBN) nanocages. In [71], the authors showed that a B_16_N_16_ fullerene-like form would have low conductivity and reactivity compared with the fullerene C_32_ and a B-doped fullerene labeled as B_8_C_24_. They concluded that the B_16_N_16_ system was the most suitable H_2_ adsorbent of these three cases.

Other theoretical calculations using DFT show the adsorption of nitrogen gas (N_2_) molecules by different fullerenes such as sphere-like C_82_ or the tubelike C_110_. The interaction between N_2_ and such fullerenes can lead to (N_2_)n molecular clusters [72]. Similar studies show that NO and NO_2_ molecules can be better adsorbed by a C_60_ fullerene when the latter is B-, N-, or BN-codoped. The best adsorption performance is obtained with the BN-codoped C_60_ fullerene, labeled as C_58_BN, and obtained after doping by substitution of two carbon atoms by nitrogen and boron atoms [73].

There are clusters formed with rare gas atoms with C_60_XN where X = Ne, Ar, Kr, or Xe. A study using an empirical approach to the potential energy surface explored the X-C_60_ and X-X interactions, showing that there is generally an energetically favorable cluster formation, with N ranging from 32 for Xe up to 60 for Ne [74]. By itself, a C_60_ fullerene can also adsorb He molecules [68].

The detection and sensing of chemical warfare agents (CWAs) is a research line where fullerenes play a relevant role. The tabun nerve agent (C_5_H_11_N_2_O_2_P), a highly toxic and dangerous gas, is an example of this, as DFT calculations have shown that a C_20_ fullerene can adsorb it. Doping the fullerene with either boron or nitrogen affects its interaction with Tabun, showing that the B-doped fullerene, or C_19_B, is better to detect the hazardous gas, due to a larger change of the system’s electrical conductivity [75].

Cyanogen (C_2_N_2_) is another toxic gas that fullerenes could detect. DFT/B3LYP calculations show that OH-functionalizing or Ge-doping of C_60_ fullerene result in a larger adsorption capacity than a pristine C_60_ fullerene, although the increase is relatively small [76]. Experimental investigations show that C_60_ fullerenes can adsorb the amphetamine (AA) drug. Additionally, DFT calculations show that the AA-C_60_ interaction increases the fullerene by substituting C atoms with either Si or Ge atoms. In both cases, the AA drug tends to be adsorbed at the (6, 6) bonds of the C_60_ structure, i.e., the bonds joining two hexagons in the fullerene structure [77].

At a larger scale, fullerenes help to detect and adsorb industrial waste. For instance, a functionalized magnetic fullerene nanocomposite (FMFNc) obtained employing a thermal decomposition of polyethylene terephthalate (PET) bottles (environmentally convenient decomposition) is an excellent candidate to purify dye-contaminated wastewater [78]. Table 3 shows the main quantitative results of this section.

### 2.4. Graphdiyne

Graphdiyne—in some works abbreviated as GDY—is a recently developed 2D material that has raised interest because it is a metal-free catalyst for low-temperature CO oxidation. It is helpful to absorb CO molecules. According to spin-polarized DFT calculations, graphdiyne can also absorb O_2_ and does it more quickly than CO adsorption [81]. However, pristine graphdiyne does not capture N_2_ efficiently [82].

In [83], the authors used DFT simulations and found that CO and methanol (CH_3_OH) are physisorbed by the pristine GDY surface, while a Ca-decorated graphdiyne (Ca-GDY) can further increase their adsorption. Ca-GDY can adsorb up to 29.81 wt% of CH_3_OH and 27.10 wt% of CO.

A GDY monolayer also adsorbs atomic oxygen and hydrogen at different sites. Hydrogen chemisorbs above a carbon atom and oxygen at the hollow place. According to such studies, oxygenated graphdiyne could be useful for spintronic devices [84].

Graphdiyne is helpful to detect amino acids, such as glycine (C₂H₅NO₂), glutamic acid (C_5_H_9_NO_4_), histidine (C_6_H_9_N_3_O_2_), and phenylalanine (C_9_H_11_NO_2_). MD simulations at room temperature suggest that graphdiyne adsorbs such amino acids with an energy more significant than that on graphene, with dispersion interactions predominating in the process [85]. Graphdiyne can also adsorb dimethylamine (DMA) and—to a lesser level—trimethylamine (TMA) vapor molecules and desorb them in a short time, making it suitable for sensing devices [86].

Other studies show that graphdiyne is a helpful drug delivery agent for medical applications. DFT and Quantum-Monte-Carlo simulations suggest that GDY adsorb the drugs sorafenib (C_21_H_16_ClF_3_N_4_O_3_) and regorafenib (C_21_H_15_ClF_4_N_4_O_3_) then release them upon protonation [87].

Graphdiyne sheets adsorb ammonia molecules (NH_3_) according to ab initio calculations, showing a better adsorption capacity than graphdiyne nanotubes [88]. Formaldehyde (HCHO) and formic acid (HCOOH) in vapor form can also be adsorbed on the top, ring and bridge sites of a graphdiyne nanosheet [89].

Functionalizing hydrogen-substituted GDY nanostructures with pyridinic nitrogen enhances its electrocatalytic performance for oxygen reduction. Hydrogen-substituted graphdiyne (HsGDY) results from bonding hydrogen to three of the C atoms in a benzene ring, facilitating nitrogen doping [90]. HsGDY and B-decorated HsGDY has also been able to physisorb several gas molecules such as NO, NO_2_, NH_3_ and N_2_. The optimal adsorption positions for these cases were located in the vicinity of the GDY benzene rings. The B-decorated HsGDY had a better adsorption performance, in particular for NO and NO_2_ [91]. Boron decoration on a regular graphdiyne layer (BGDY) has also been considered as a possible electronic sensor for anticancer drugs like temozolomide (TMZ). The B decoration was also shown to increase the TMZ adsorption capabilities of GDY, related to an increase in its electrical conductivity of around 40%, according to ab initio calculations [92]. The 5-fluorouracil (5FU) anti-cancer drug—which shows no interaction with pristine GDY—can also be adsorbed by a BGDY composite, increasing the latter’s electrical conductivity by around 25%, a feature that would make BGDY suitable for 5FU sensing [93].

Besides boron, other nonmetal atoms like Si, P, S, As, Se, and Te are used through DFT calculations to decorate graphdiyne surfaces to increase their ability to dissociate molecular oxygen. The As- Se- and Te-decorated GDY monolayers physisorbed the O_2_ molecule, while the B, N, O, Si, P, and S-decorated GDY chemisorbed it [94].

Li, an alkali metal, is another exciting option. The authors in [95,96] showed that this metal catalyzes the adsorption properties of graphene or carbon nanotubes. Studies have compared DFT-PBE and hybrid DFT+LC-⍵PBE calculations to explore the Li adsorption on graphdiyne. PBE tends to overestimate the adsorption energy, suggesting that hybrid DFT should be preferred when studying GDY electronic properties [97]. Similar studies, including Li-decorated GDY, or Li@GDY, show that it could capture CO_2_ molecules while investigating the possible conversion of CO_2_ into beneficial—or at least less nocive—chemicals via carbon dioxide electrochemical reduction reaction (CO_2_RR) [98].

In [99], the authors found that CO_2_RR improved with nitrogen doping on graphdiyne (NGDY), increasing the selectivity regarding the doping with CH_3_OH and CH_4_. In particular, the lowest limiting potential of CH_3_OH on NGDY gets reduced. Further composites such as boron- and nitrogen-doped GDY anchoring a single Cu atom (labeled as Cu@N- and Cu@B-doped GDY) have also been systematically explored via ab initio calculations. The result is that Cu@N-doped GDY monolayers are more efficient than the boron-doped ones for CO_2_ reduction and highly catalytic activity toward CO_2_RR [100].

However, there are cases where boron-doped GDY shows a better performance than nitrogen-doped GDY, as demonstrated by spin-polarized DFT studies. For instance, B-GDY shows excellent sensitivity and selectivity toward NO, NO_2_, and ammonia (NH_3_) [101]. Recently, a DFT study combining boron and nitrogen doping—a BN co-doping labeled as BN@GDY—of defective graphdiyne showed that it could increase the catalytic efficiency compared with boron doping alone. The increment in catalytic efficiency would be by a change in the B hybridization from sp^2^ to sp^3^ caused by the introduction of N atoms [102].

Improving catalysis can be seen the other way around: instead of decorating graphene sheets, they could be the decoration. For instance, when placed on the Pt(111) surface, a graphdiyne layer gives better results at increasing the latter two-dimensional confined catalysis performance compared with graphene or hexagonal boron nitride [103].

Finally, an increasing interest in the effective detection of chemical warfare agents (CWAs) has led to the investigation of graphdiyne as a possible sensor. Using ⍵B97XD DFT and Quantum theory of atoms in molecule (QTAIM) analysis, studies in [104] found that G-type nerve agents like GA(tabun), GB(sarin), GD(soman), and GF(cyclosarin) can be physisorbed at the triangular section of a GDY nanosheet. The GDY surface shows a short recovery time at room temperature, suggesting it can be a good option for sensor devices. Highly nocive A-series CWAs can also be physisorbed by GDY nanoflakes via noncovalent adsorption, as per DFT-⍵B97XD calculations that consider long-range interactions [105]. Lewisite molecules are another type of toxic CWAs that DFT calculations show can be adsorbed by graphdiyne nanoflakes. In this case the study shows that GDY physisorbs Lewisite L_1_, L_2_ and L_3_ with the highest GDY sensitivity being found to be towards L_2_ [106]. Table 4 shows the main quantitative results of this section.

### 2.5. Hybrid Systems

We define a hybrid system as the surface created using different carbon nanostructures, i.e., the combination of graphene or graphdiyne with nanotubes or fullerenes or semi fullerenes or buckybowls. The result is a set of structures with a larger specific area for gas adsorption purposes. For instance, theoretical investigations use DFT focused on the hydrogen adsorption on a system made of graphene decorated with C_176_. The results show that the adsorption energies ranged between 0.069 and 0.115 eV. Up to four hydrogen molecules could get into the C_176_ [109].

Another frequently investigated structure for gas adsorption is pillared graphene, which comprises graphene sheets joined by carbon nanotubes. In [110], the authors investigated the hydrogen storage capacity in pillared graphene using MD calculations to study the effects of the environment’s pressure, temperature, and geometric structure. They found that pillared graphene has a higher performance than CNTs as a hydrogen storage material, a fact that due to its larger surface area.

Using a hybrid molecular dynamics–Monte Carlo simulation method, the authors in [111] investigated the methane (CH_4_) adsorption on Pillared graphene. They found that in this case, the CNT length has the most significant effect on the adsorption amount of methane among different geometrical parameters such as CNT diameter, graphene sheet layer spacing, and the number of CNTs. The adsorption ability of pillared graphene is greater than that of graphene sheets. Such a structure has higher mechanical stability than graphene.

A variation of the pillared graphene system considers fullerenes instead of nanotubes placed between the graphene sheets. In [112], the authors considered this variation and studied methane adsorption with adjustable micro and mesoporous morphology. Their simulations via grand canonical Monte Carlo simulations revealed that this system is suitable for methane storage. Other studies performing similar simulations have explored the same kind of nanostructure for hydrogen adsorption [113]. They considered three different fullerenes such as C_180_, C_320_, and C_540_. Hence, this would be a potentially helpful system for hydrogen storage.

Other studies involving grand canonical Monte Carlo simulations considered fullerenes placed between graphene sheets and lithium doping to investigate methane (CH_4_) adsorption. These structures are labeled as Sandwiched Graphene-Fullerene Composite (SGFC) and have a promising potential for methane storage applications. This Li-doped nanostructure could be considered suitable host materials for lightweight methane storage devices [112]. The same SGFC, both undoped and Li-doped, has been explored in terms of its hydrogen adsorption capacity. Employing grand canonical Monte Carlo calculations, three fullerene types as the sandwich core have also been studied: C_180_, C_320_, and C_540_. It was observed that a Li-doped nanostructure with a doping ratio of Li:C = 1:8 can overpass the gravimetric capacity of 5%, while an undoped one can reach the value of 3.83% at 77 K and 1 bar. The Li-doped SGFC capacity is larger than that observed in graphene-based systems [113].

Carbon nano onions (CNO) are another interesting mixed nanostructure discovered in 1992. These systems are multi-shell fullerenes with concentric shells of carbon atoms with an interlayer distance similar to graphite [114]. Experimental investigations on the synthesis of hollow carbon nano-onions and their use for adsorbing hydrogen molecules showed that the hollow CNOs exhibit good performance as materials for electrochemical hydrogen storage. Furthermore, using DFT calculations, the authors in [115] studied the properties of stoichiometric and defective nitrogen carbon nano onions, or NCNOs. Exploring the stability and thermodynamic properties of mono and divacancy-containing CNOs showed that the latter is more stable and can increase the bandgap notably.

To finish this section, we will focus on buckybowls. A buckybowl is not a hybrid system, but an interesting one nevertheless. These structures come from the fragmentation of fullerenes. Buckybowls are adsorbents of CO_2_, CH_4_, and C_2_H_2_. Studies using hybrid DFT calculations found that the curvature of the buckybowl surface plays a significant role in the adsorption of these gas molecules. Buckybowls are high-performance materials for capturing greenhouse gases, molecular recognition, and nanotechnology applications in general [116]. Hybrid nanostructures can also see their adsorption capacity increase by doping them, usually with lithium and nitrogen as dopants. Table 5 shows the main quantitative results of this section.

## 3. Adsorption on Systems Doped with Transition Metals

### 3.1. Transition Metals

The definition of transition metals usually refers to those elements in groups 3–12 on the periodic table, with partially filled d-orbitals. The frequent name for the lanthanide and actinide series is inner transition metals. However, it is more frequent to call transition metals the elements of groups 4–11. This fact relies on the typical chemical behavior of the metals in these groups. Note that the filling of the d-orbitals increases going from left to right in the periodic table. It is usual to name them the “d-block elements.” Thus, these metals have several properties in common. First, they have low ionization energies, and several of them form paramagnetic compounds. Second, these metals are reactive but not as much as the alkalis. Finally, they conduct heat and electricity very well and have high melting and boiling temperatures.

### 3.2. Nanotubes Doped with Transition Metals

Doping nanotubes to increase their adsorption capacity is frequent. In particular, the decoration involving transition metals, oxides, or polymers, can increase their sensitivity [119]. It is also frequent to use transition metals Pd, Pt, Ag, Au, and Rh for doping carbon nanotubes that in turn can be used as gas sensors [31,120,121,122,123,124,125,126,127,128,129,130,131,132,133,134,135,136] for detection of gases of C_3_H_6_O (acetone), C_6_H_6_ (benzene), H_2_, CH_4_, NH_3_, NO_2_, H_2_O, CO, H_2_S, HCN, HCl, Cl_2_. Specifically, Pd used for the adsorption of H_2_, Cl, CH_4_, Cl_2_, acetone, benzene; Pt used for the adsorption of C_7_H_8_ (toluene), H_2_, NO_2_, H_2_O, NH_2_; Au for the adsorption of C_2_H_6_O (ethanol), NH_3_, NO_2_, and Rh used for the adsorption of HCl, CO, and CH_4_. Furthermore, doping with Fe makes adsorption of the hydrogen molecule stronger than on an undoped nanotube. The adsorption of Fe on the nanotube is exothermic [137].

Experimental studies found that the H_2_ storage capacity of pristine MWCNTs increases up to 15 times at room temperature and 2.0 MPa by decorating them with ultrafine Ti nanoparticles. This improvement does not affect the cyclic hydrogenation–dehydrogenation operation of the tubes [138]. The intercalation of nickel on MWCNTs increases the H_2_ adsorption, probably due to the strong interaction between the Ni atoms and the MWNTs [139].

DFT calculations of Pt-decorated nanotubes show different effects in the adsorption capacities of the latter. For instance, the nanotube conductivity can vary with the adsorption of H_2_S, CO, and SO_2_ [140]. Another DFT calculation shows that Au-decorated nanotubes have a high sensitivity to adsorption of SO_2_ and H_2_S [141]. These Au-decorated nanotubes help sense propanone or acetone (C_3_H_6_O) gas, with the Au decoration increasing the device sensitivity up to around 3% at room temperature [142].

Rh-doped single-walled carbon nanotubes are advantageous for the adsorption of CO, C_2_H_4_ (ethylene), and SO_2_ molecules while proving to be insensitive to CO_2_ and CH_4_ [143]. DFT calculations using dispersion corrections in [144] showed that the doping of a (14,0) carbon nanotube by substituting a carbon atom with a Ru atom conveys better adsorption of SO_2_ and H_2_S molecules by the Ru-doped SWNT.

Pd and Pt decorations on a nanotube are practical to adsorb CO and NO molecules. Pd decoration is more suitable to adsorb NO, while Pt works better for CO adsorption. Ab initio calculations showed that this adsorption would change the magnetic properties of the Pd- and Pt-decorated SWCNTs, which would be helpful for sensors [145].

Pd as a dopant facilitates the adsorption of N_2_O on the otherwise inert nanotube [146]. More DFT calculations predict that Rh-doped nanotubes present a good adsorption ability of O_2_ and O_3_ and show relatively significant conductivity changes [147]. There is experimental evidence that Au nanoparticles adsorbed on nanotubes are helpful for acetone gas sensing at room temperature [148].

Table 6 presents a list of transition metals used for doping carbon nanotubes and the gases adsorbed in the doped nanotube. Notice that the most used is Au, and the least used are Ag and Fe. From the 32 elements in the periodic table (groups 4–11), we found mainly six transition metals used as dopants. Table 7 shows the main quantitative results of this section.

### 3.3. Graphene Doped with Transition Metals

We label TM-doped graphene the graphene doped with transition metals in this review. As with the other nanostructures considered in this review, hydrogen adsorption is a heavily explored topic, and transition metal-doped graphene has been no exception. For instance, a TiO_2_ decoration of nanoparticles on graphene oxide (GO + Ti) can adsorb twice as much H_2_ as pristine graphene at room temperature and 5 MPa, showing reversibility of up to 80% [149]. Copper-decorated N-doped defective graphene nanoribbons can improve their reactivity towards H_2_ adsorption. In [150], a DFT study considered single vacancy defects on graphene doped with N atoms, forming a pyridine-like structure. The authors labeled these structures as SV+1N, SV+2N, or SV+3N, depending on the number of nitrogen atoms. They added Cu decoration on each resulting system and concluded that the Cu-decorated SV+3N showed the best H_2_ adsorption performance, with a promising reversible cycle.

TM-doped graphene has been considered a possible candidate to sense different polluting gases, which usually interact weakly with pristine graphene. We have the chemical warfare agents (CWAs) among the pollutant gases under investigation, mentioned in Section 2.3. Transition metals such as Zr, Mo, Ti, Mn, Fe, and Co dope graphene and increase adsorption capabilities. For instance, Mn-doped graphene shows the most substantial chemical adsorption of phosgene (COCl_2_), a highly toxic CWA [59].

Other DFT studies suggest that Mn-doped graphene substantially improves the adsorption of C_2_H_2_, CH_4_, and CO, which are characteristic dissolved gases in transformer oil. In [54], the authors found that this substrate would be appropriate for sensing only the first two gases mentioned. The adsorption of CH_4_ is weak due to an electronic hybridization.

TM-doped graphene with TM = Ni, Cu, Zn, or Ni-doped vacancy defect graphene (DG) and graphene oxide (G-OH) adsorb hydrogen sulfide (H_2_S) that is another toxic and colorless gas. DFT studies show that Cu-doped graphene and DG display the best performance to adsorb H_2_S among the cases considered. Ni and Zn-doped graphene show weaker adsorption energies. Other studies show that Pt-decorated graphene is an H_2_S detector. The most stable configuration for adsorption corresponds to the H atoms of the H_2_S pointing towards the TM-doped graphene [46,48,58]. The bilayer graphene (BG) adsorbs H_2_S, and this adsorption increases by doping (BG) with transition metals as Fe, Ni, Mn, Cr, Co, and V. Notice that the TM-doping can occur at more sites concerning a single graphene layer, for example, in the interlayer region.

DFT calculations also show that an external electric field would further increase the adsorption of H_2_S onto such TM/BG systems. As most TM/BG-H_2_S systems have semiconductor behavior, this could also be a good candidate for sensing devices [53]. Additionally, the H_2_S sensing performance of zigzag graphene nanoribbons (ZGNR) is also increased mainly by combined doping of Cu and Zn atoms, the resulting system labeled as Cu/Zn-ZGNR, with a high response value of around 49% [51].

TM-doped graphene (with TM = Ti, Mn, Fe, Co, Ni, and Ag) adsorbs arsine (AsH_3_), another toxic gas. As in previous cases, TM doping increases the chemical interaction with the gas compared with pristine graphene. In [57], the authors found that Ni-doped graphene has the best AsH_3_/CO selectivity, an important feature when removing arsine from CO gas streams. The resulting hybridization of states between AsH_3_, CO, and TM orbitals confirmed the chemical interaction.

Recent theoretical investigations utilizing DFT considered Ag- Pt- and Au-doped graphene as a possible sensing material of nitrogen oxides such as NO and NO_2_. Au-doped graphene shows the best adsorption performance of NO/NO_2_ molecules of the three transition metals considered. Ag/Pt/Au-doping generally gives better sensing results than non-TM doping like B, N, Al, or S [56]. In [151], from DFT simulations, the authors found that Fe-doped graphene shows a high NO_2_ adsorption rate due to the d-orbital impurity states from the metal on the graphene surface. They also found that an increase in the strain applied to the graphene sheet decreases its adsorption capacity. Fe-doped armchair graphene nanoribbons (Fe-AGNR) are candidates for NO and NO_2_ sensing devices due to their electronic and transport properties [152].

Group 10 transition metals such as Ni, Pd and Pt can also decorate graphene to increase its interactions with NO_2_ [46] significantly. Of the same group 10, palladium is another exciting option for graphene doping. It has been shown via DFT calculations that Pd-doped graphene can adsorb CO and NO molecules, with the most stable site being a bridge site (B-site). Pd doping vastly increases the interaction between the substrate and CO/NO [55].

Platinum clusters like the Pt_13_ structure, when supported on pristine graphene, can also enhance the adsorption capacity of the latter. A systematic DFT study showed that such a composite could adsorb CO_2_, NO_2_, and SO_2_ more effectively than defective graphene (DG) supported Pt_13_ [153].

The latter gas, sulfur dioxide, has also been the subject of experimental and theoretical studies involving TM-decorated graphene. SO_2_ can be adsorbed more efficiently by metal-oxide such as ZnO, BeO, and Ni-decorated graphene than pristine graphene. ZnO- and BeO-decorated graphene physisorb SO_2_, while Ni-decorated graphene chemisorbs it [52].

TM-doped graphene can also enhance the detection of formaldehyde (HCHO) gas. A first-principles study considered a two-probe sensor device built with TM-doped graphene, where TM = Co, Ni, Cu, Zn, Pd, and Ag. The Cu- and Ag-doped graphene devices showed the best performance at low voltages, with a short response time and high HCHO sensing [154]. Table 8 shows the main quantitative results of this section.

### 3.4. Fullerenes Doped with Transition Metals

The TM-doping or decoration increases the adsorption capacities of fullerenes. This increase by doping is present in the other carbon nanostructures reviewed in this work. In [79], the authors performed a systematic DFT study focused on TM decoration of C_60_ fullerene, with TM = Ti, V, Cr, Mn, Fe, Co, Ni, Cu, and Zn. The changes in the electronic structure implied that the TM-decorated C_60_ fullerene is more sensitive to CO and NO molecules than the pristine one.

Doping fullerenes with titanium increases their H_2_ storage capacity. A way of doing it is by substituting carbon atoms with titanium atoms and doping a C_60_ fullerene with an increasing number of Ti atoms, from one up to six. With six Ti atoms doping C_60_ fullerenes in this fashion—labeled as Ti_6_C_48_—the hydrogen storage can be up to 7.7 wt% [156]. In [157], the authors performing DFT simulations found that Co atoms forming compact clusters on the surface of a C_60_ fullerene labeled as C_60_Co_n_, chemisorb an H_2_ molecule, with different features for different values of *n* (*n* = 1 to 8). They also found that up to 13 H_2_ molecules could be absorbed in this way.

Theoretical calculations performed in [158] showed that a C_70_ fullerene doped with TM ions (Cr^2+^ and Co^2+^) adsorbed nitrogen dioxide (NO_2_) on the exterior surface. The doping mechanism is a porphyrin-induced process labeled as PIC70F. In this manner, this fullerene would be helpful for selective detection of NO_2_ in the presence of sulfur dioxide, also showing a short recovery time.

Another way of doping fullerenes is from the inside, an example of which would be the endohedral metallofullerenes (EMFs). Such structures are obtained by putting a lowest energy structure of a metal cluster inside of the fullerene and then relaxing the system. When C_60_ fullerenes are considered, the EMFs obtained in this manner are labeled as Mn@C_60_, and they can increase the oxygen reduction reaction (ORR) activities of the former. A comprehensive DFT study showed that EMFs such as Mn_5_@C_60_, Cu_4_@C_60_, Co_2_@C_60_, and Ni_4_@C_60_ have better ORR activities, which could be helpful in developing sensing devices able to detect polluting gases [159]. Table 9 shows the main quantitative results of this section.

### 3.5. Graphdiyne Doped with Transition Metals

Transition metals (TMs) can enhance the adsorption properties of graphdiyne (GDY). Several DFT studies have explored the adsorption of metals like Au, Cu, Ni, and Zn—although Zn is not a transition metal—on graphdiyne. GDY strongly chemisorbs nickel, and it also chemisorbs silver and copper. On the other hand, graphdiyne physisorbs a zinc atom. Overall, this material is better adsorbent for those metals compared with graphene [107]. When the GDY surface first adsorbs one or two nitrogen atoms, the resulting N-GDY composite increases the stability of further transition metal decorations. In [160], the authors investigated the adsorption of several TMs (Cr, Mn, Fe, Co, Ni, and Cu) on such N-GDY substrates. They found that Fe@2N-GDY (a Fe atom decorating a GDY doped with 2 N atoms) has the best catalytic activity with the lowest CO oxidization energy barrier.

As with the other nanostructures covered in this review, graphdiyne has been explored for its H_2_ adsorption capabilities, aiming at reversible hydrogen storage. DFT studies have considered decorating graphdiyne nanosheets with light metals such as Li, Na, K, Ca, Sc and Ti. Some works have explored up to 11% of metal functionalization in the GDY surface, which can then anchor multiple H_2_ molecules [161].

Water splitting is another exciting application of TM-decorated GDY nanosheets. For instance, theoretical simulations and experimental studies show that Ru-doped GDY (Ru/GDY) has a high catalytic performance for oxygen evolution reactions [162].

The electrochemical reduction of N_2_ at room temperature is relevant for the production of ammonia (NH_3_). A systematic study considering transition-metal-embedded GDY, or TM@GDY, explored the effect of Sc, Fe,Cr, Mn, Mo,Co, Ni, Cu, Zn, Ru, Rh, Pd, and Ag in the N_2_ reduction reaction. According to spin-polarized DFT simulations, the most stable and best catalytic activity was the Mo-embedded graphdiyne monolayer [163]. All TMs increase GDY sheets’ N_2_ reduction reaction (NRR) character. However, other studies indicate that the single-atom catalyst V@GDY monolayer has the best NRR performance [82].

Anchoring two transition metals to GDY improves the NRR activity. In a study involving DFT calculations, the authors considered several TMs (Fe, Co, Ni, Cu, and Mo). They found that the best NRR performance was with the Co-Ni heteronuclear complex (CoNi@GDY) and the Mo-Mo homonuclear complex (Mo_2_@GDY) [164].

Scandium and titanium can also decorate graphdiyne to enhance sensing of formaldehyde (HCHO), a typical air pollutant. Sc or Ti take a stable place on the corner sites of graphdiyne sheets, and both can better adsorb HCHO compared with pristine graphdiyne and graphene. This effect would be due to the electronegativity of HCHO, and the best result is with Ti-decorated graphdiyne, according to DFT calculations [165].

In [108], the authors investigated the palladium clusters supported on graphdiyne surfaces utilizing DFT calculations. They found that the electronic HOMO-LUMO gap changes with the size of said clusters, which chemisorbed with relatively high adsorption energies of around 3–4 eV in magnitude. Palladium nanoparticles can also be experimentally anchored to the GDY surface, forming a stable nanocatalyst labeled PdNPs/GDY. Such composite can decompose H_2_O_2_ to produce O_2_, an essential reaction in antitumor treatments combined with doxorubicin, a chemotherapeutic agent [166].

Doping GDY with Fe atoms improves the electrochemical reduction of CO_2_ by GDY sheets. An ab initio study found that doping the GDY surface with a Fe dimer or a Fe trimer optimizes the CO_2_ adsorption and selectivity. The number of Fe atoms considered can generally tune the GDY catalytic activity significantly [167]. CO oxidation would also be accomplished employing several single-atom catalysts (SACs) such as Ni-GDY and Cu-GDY. DFT calculations have shown that both Ni and Cu can be anchored at the corner of the acetylenic ring of graphdiyne, the resulting system being able to adsorb CO [168]. Table 10 shows the main quantitative results of this section.

### 3.6. Hybrid Systems Doped with Transition Metals

Several studies have also focused on the TM-doping of hybrid carbon nanostructures starting. The most convenient places to dope a buckybowl have been studied by computational means, covering the binding possibilities of the transition-metal ion Cu^+^, and cations as Li^+^, Na^+^, K^+^, to buckybowls on convenient locations where the gradient electron density favors the adsorption [169]. In other studies, authors have investigated the catalytic effect of Ni, Fe, and a Fe-Ni alloy on synthesizing metal-containing carbon nano-onions (CNOs) and studied their electrochemical hydrogen storage properties. They found that the electrochemical hydrogen storage capacity of the CNOs is in the order of Ni@CNOs > Fe_0.64_Ni_0.36_@CNOs > Fe_3_C@CNOs. The Ni@CNOs have a maximum hydrogen storage capacity of 1.42%. Large amounts of defects, good electrical conductivity, and electrocatalytic activity of the Ni particles are responsible for their excellent electrochemical performance [170].

In [171], the authors considered the doping of a semi-fullerene C_30_ with titanium to adsorb molecules of CO and CO_2_. With an exploration involving density functional theory (DFT) and first-principles molecular dynamics (FPMD) at 300 K and atmospheric pressure, they found that the most stable adsorption of the titanium atom on C_30_ occurs on the concave surface of the molecule. Besides, the considered molecules are chemisorbed, with no dissociation. The adsorption energies depend on the initial orientation of the molecules concerning TiC_30_. Similarly, in [172], density functional theory (DFT) was also used to study the adsorption of an H_2_ molecule in a system formed by a graphene layer and a Ti-doped semi-fullerene. The authors found that the semi-fullerene is bound to the graphene layer, with one of the hexagonal faces of the former being oriented into the latter. Besides, the semi-fullerene chemisorbs the titanium atom. Finally, the authors studied the interaction between the hydrogen molecule and the combined system, finding that the system can adsorb the H_2_ molecule. Table 11 shows the main quantitative results of this section.

## 4. Conclusions

We aimed to present the most important and recent advances in the study of polluting gas adsorption employing carbon nanostructures. The porous nature of the latter has made them a natural choice for developing sensing devices throughout the years, and physical features like diameter, curvature or size, influence their adsorption capabilities. The carbon nanostructures reviewed in general physically adsorb many of the pollutant gas molecules considered, and the interaction can usually increase by different means of nonmetal functionalizations or doping. However, transition-metal doping and decoration give better results overall, increasing their sensing properties, involving chemisorption in most cases studied. There have also been many systems showing a good adsorption–desorption cyclic performance, which makes them good candidates for sensing devices.

Among the transition metals used as dopants in the reviewed works, we found Ag, Au, Pt, Pd, Fe, Rh, Zr, Mo, Ti, Mn, Co, Ni, Cu, Sc, and V. Besides, we found the non-transition metals as dopants too: Zn, Si, P, S, As, Se, Te, Li, N, and B.

On the other hand, among the pollutant molecules investigated for adsorption in carbon nanostructures that we found in this review, we have NO_2_, NH_3_, NH_2_, SO_2_, H_2_S, C_2_H_6_O, C_3_H_6_O, C_7_H_8_, NO, Cl_2_, CH_4_, N_2_O, CO, C_2_H_4_, CO_2_, O_3_, CH_3_OH, H_2_CO. H_2_CO_2_, C_2_H_2_, SF_6_, C_2_N_2_, C_6_H_14_, C_7_H_8_, C_2_H_3_N, CH_2_Cl_2_ CH_3_COOC_2_H_5_, (CH_3_)_2_NH, C_3_H_9_N, AsH_3_. Moreover, the anticancer drugs 5-fluorouracil and temozolomide. We also found amphetamines. Finally, we also found the chemical warfare agents (CWAs) tabun, sarin, soman, cyclosarin, phosgene, and Lewisite molecules.

Carbon nanotubes and graphene are probably the most investigated systems, as shown by the abundance of cites. However, the detailed study of its adsorption properties is ongoing.

To end, we should mention that the study of the adsorption properties of carbon nanostructures is far from being completed. Relatively recent structures like graphdiyne have plenty of potential applications waiting for the investigation, and the building of hybrid systems has much to offer for materials scientists in the future.

## Figures and Tables

**Table 1:** Summary of adsorption energies E_ads_ (in eV) for different non-metallic doped nanotubes systems and adsorbates. The type of study is also specified.

System	Type of Study	Adsorbate	E_ads_
CNTs [41]	DFT	NO_2_ CH_4_ CO_2_	−0.427 −0.122 −0.109
SWCNTs [39]	GCMC, MD	SO_2_	−0.464
MWCNTs [11]	Experimental	H_2_	[−0.26, −0.046]
MWNTs [28]	Experimental	SF_6_	[−0.529, −1.285]
SWCNTs [37]	DFT	NO_3_	−1.30
CNNT [38]	DFT	H_2_CO	−0.321
SWNTs and bundles [10]	DFT	NO_2_ O_2_ H_2_O NH_3_ N_2_ CO_2_ CH_4_ Ar	−0.427 −0.306 −0.128 −0.162 −0.123 −0.109 −0.122 −0.082
SWCNTs [12]	Experimental	H_2_	−0.056
SWCNTs and SiC-DC [27]	GCMC	CO_2_ CH_4_	−0.005 −0.003
SWCNTs [30]	Exp. and DFT	C_3_H_6_O	[−0.255, −0.771]
CNTs [31]	Experimental	C_2_H_5_OH	Not reported
CNTs films [19]	Exp. and GCMC	CO_2_	Not reported
MWCNTs [40]	Experimental	VOC	Not reported
SWCNTs [36]	Exp., GCMC, MD	Xe	Not reported
F-MWCNTs [24]	Experimental	CO_2_	[−0.084, −0.036]
F-MWCNTs [13]	Experimental	H_2_	Not reported
Oxygen F-CNTs [26]	Experimental	C_2_, N_2_	Not reported
PANI/MWCNTs [32]	Experimental	NH_3_	Not reported
F-MWCNTs and A-MWCNTs [14]	Experimental	H_2_	Not reported
1,3-diaminopropane MWCNTs [22]	Experimental	CO_2_	Not reported

**Table 2:** Summary of adsorption energies E_ads_ (in eV) for different non-metallic doped graphene systems and adsorbates. The type of study is also specified.

System	Type of Study	Adsorbate	E_ads_
ZGNR [51]	DFT	H_2_S	−0.364
Pristine G (PG) [52]	DFT	SO_2_	−0.157
PG [49]	Exp. and MD	NH_3_	Not reported
PG [44]	DFT and MD	H_2_	Not reported
Graphene [48]	DFT	H_2_S CH_4_	−0.038 −0.022
DG (vacancy) [48]	DFT	H_2_S CH_4_	−2.934 −0.154
G-OH [48]	DFT	H_2_S CH_4_	−1.263 −0.047
Single layer G (SLG) [43]	Experimental	H_2_	Not reported
Bilayer G (BG) [53]	DFT	H_2_S	−0.360
PG [54]	DFT	CH_4_ C_2_H_2_ CO	−0.086 −0.102 −0.093
PG [46]	DFT	NO_2_ H_2_S	[−0.214, −0.185] [−0.201, −0.122]
PG [55]	DFT	CO NO	−0.084 −1.166
Vacancy G (VG) [55]	DFT	CO NO	−0.069 −1.203
PG [56]	DFT	NO NO_2_	[−0.1280, −0.1176] [−0.1679, −0.1427]
PG [57]	DFT	AsH_3_ CO	Not reported
PG [58]	DFT	H_2_S	−0.360
PG supercell [47]	DFT	NO	[−2.3713, −1.7453]
PG [59]	DFT	COCl_2_	−0.554
G nanoflakes (GNFs) [60]	DFT	CO CO_2_	−1.18 −0.58
PG [45]	DFT	H_2_O NH_3_ CO NO_2_ NO	−0.047 −0.031 −0.014 −0.067 −0.029
PG [42]	DFT	H	[−0.84, −0.75]

**Table 3:** Summary of adsorption energies E_ads_ (in eV) for different non-metallic doped fullerene systems and adsorbates. The type of study is also specified.

System	Type of Study	Adsorbate	E_ads_
C_60_ [70]	Exp. and DFT	H_2_	[0.0495, 0.0641]
C_60_ [74]	PES (potential energy surface)	Ne, Ar, Kr, Xe	Not reported
C_60_ [69]	Experimental	H	−0.0247
C_60_ [72]	DFT	N_2_	[−0.28, −0.03]
C_60_ [64]	GCMC	C_2_H_4_	[−0.0207, 0.0207]
C_60_ [79]	DFT	CO NO	−0.006 0.00008
C_60_ [76]	DFT	C_2_N_2_	−4.78
OH-C_60_ [76]	DFT	C_2_N_2_	−5.13
C_60_ [77]	DFT	C_9_H_13_N + N C_9_H_13_N + H	−0.017 −0.0516
C_32_ [71]	DFT	H_2_	[−0.118, −0.0086]
C_60_ [65]	Experimental	Aromatic vapor	Not reported
C_60_-N_x_ [73]	DFT	NO NO_2_	[−0.35, −0.28] [−1.01, −0.94]
C_24_ + P + N_24_ [80]	DFT	CO_2_	[−0.94, −0.30]
C_60_+P [63]	DFT	CO_2_	[−1.97, 0.06]
Si@C_54_N_4_ [66]	DFT	N_2_O CO O_2_	[−3.50, −0–77]
C_60_ [67]	DFT	N_2_O CO	−0.169 −0.092
C_20_ [75] B-C_20_ [75] N-C_20_ [75]	DFT	C_5_H_11_N_2_O_2_P	[−0.054, −0.043] [−1.092, −0.072] [−0.027, −1.65]
C_460_ [62]	DFT	H_2_ CO_2_	−4.37 [−0.49, −0.42]
C_60_ [68]	Modified LJ-potential	H_2_, He	Not reported

**Table 4:** Summary of adsorption energies E_ads_ (in eV) for different non-metallic doped graphdiyne systems and adsorbates. The type of study is also specified.

System	Type of Study	Adsorbate	E_ads_
GDY [86]	DFT	DMA TMA	[−0.503, −0.757] [−0.607, −0.796]
GDY supercell [85]	DFT	C_2_H_5_NO_2_ C_5_H_9_NO_4_ C_6_H_9_N_3_O_2_ C_9_H_11_NO_2_	[−1.10, −0.59] [−1.14, −0.54] [−1.46. −0.73] [−1.53, −0.77]
GDY, BGDY [92]	DFT	TMZ	[−1.97, −0.95]
GDY nanoflakes [105]	DFT	CWA A-230 CWA A-232 CWA A-234	−0.594 −0.713 −0.745
GDY [81]	DFT	CO O_2_	−1.43 −3.27
GDY-NS [87]	DFT	C_21_H_16_ClF_3_N_4_O_3_ C_21_H_15_ClF_4_N_4_O_3_	[−0.660, −0.085] [−0.641, −0.081]
GDY [89]	DFT	CH_2_O CH_2_O_2_	[−1.502, −0.342] [−0.945, −0.390]
GDY [107]	DFT	Ag Cu Ni Zn	[−0.792926, −1.236] [−0.622651, −2.783] [−2.913467, −3.446] [−0.0196, 0.0356]
GDY [84]	DFT	H O	−3.73 −7.53
GDY nanoflakes [106]	DFT	L_1_ L_2_ L_3_	−0.441 −0.534 −0.567
GDY [97]	Hybrid DFT	Li	−1.82
Ca-GDY [83]	DFT	CH_4_O CO	[−0.349, −0.122] [−0.128, −0.060]
GDY [104]	DFT, QTAIM	CWA GA CWA GB CWA GD CWA GF	−0.707 −0.520 −0.543 −0.382
GDY [88]	DFT	NH_3_	[−0.465, −0.435]
GDY [108]	DFT	Pd clusters	[−4.0, −3.0]

**Table 5:** Summary of adsorption energies E_ads_ (in eV) for different non-metallic doped hybrid systems and adsorbates. The type of study is also specified.

System	Type of Study	Adsorbate	E_ads_
CNO [117]	Experimental	H_2_O	Not reported
CNO [115]	DFT	mN, m = 1–10	[−0.18, 0.33]
Buckybowls [116]	Hybrid-DFT	CO_2_, CH_4_, C_2_H_2_	Not reported
C_20_, C_20_ (bowl) [118]	DFT	N, H	Not reported
Nanobuds [109]	DFT	H_2_	[0.069, 0.115]
Sandwiched G-fullerene + Li [113]	GCMC	H_2_	Not reported
Pillared-graphene [110]	MD	H_2_	Not reported
Fullerene pillared-graphene [112]	GCMC	CH_4_	Not reported
Pillared-graphene [111]	GCMC-MD	CH_4_	Not reported

**Table 6:** List of transition metals used for doping carbon nanotubes and the corresponding adsorbed gases.

Metal	Adsorbed Molecules
Ag	NO_2_ [133]
Au	NH_3_, NH_2_ [132,133,135]; SO_2_, H_2_S [141]; C_2_H_6_O [31]; C_3_H_6_O [142,148]
Pt	H_2_, NO_2_, H_2_O, NH_3_ [132]; C_7_H_8_ [136]; NO [145]
Pd	H_2_ [120,121,122,123,124,125,128]; NO_2_ [135], Cl_2_ [135]; CH_4_ [126,127]; NO [145]; N_2_O [146]
Fe	H_2_ [137]
Rh	CO [134]; CO, C_2_H_4_ SO_2_ [143]; O_2_, O_3_ [147]

**Table 7:** Summary of adsorption energies E_ads_ (in eV) for different TM-doped nanotubes systems and adsorbates. The type of study is also specified.

System	Type of Study	Adsorbate	E_ads_
CNT(APTS) [18]	Experimental	CO_2_	Not reported
Fe-SWCNTs [137]	DFT	H_2_	[−0.238, −0.141]
Au-CNTs [31]	Experimental	C_2_H_6_O	Not reported
Pt-SWCNTs [140]	DFT	SO_2_ H_2_S CO	−1.225 −0.977 −1.386
Au-SWCNTs [141]	DFT	SO_2_ H_2_S	−1.258 −1.317
Au-CNTs [142]	Experimental	C_3_H_6_O	Not reported
Rh–CNT [143]	DFT	CO CO_2_ CH_4_ C_2_H_4_ SO_2_	[−3.527, −1.308] −0.348 −0.253 −1.189 −1.158
Pd-, Pt-SWNTs [145]	DFT	CO NO	[−1.8, −1.6] [−1.814, −1.46]
Pd-CNT [146]	DFT	N_2_O	−0.91
Rh–CNT [147]	DFT	O_2_ O_3_	−1.384 [−2.711, −1.824]
Pt-MWCNTs [136]	Experimental	C_7_H_8_	Not reported
Au-CNTs [148]	Experimental	C_3_H_6_O	Not reported
Pd-, Pt-, Rh-, Au-SWNTs [134]	Experimental	H_2_, CH_4_, CO, H_2_S	Not reported

**Table 8:** Summary of adsorption energies E_ads_ (in eV) for different TM-doped graphene systems and adsorbates. The type of study is also specified.

System	Type of Study	Adsorbate	E_ads_
Zn-ZGNR [51]	DFT	H_2_S	−2.237
Cu-ZGNR [51]	DFT	H_2_S	−1.129
Cu/Zn-ZGNR [51]	DFT	H_2_S	−7.043
Ni-G [52]	DFT	SO_2_	−2.297
TM-PG [154] TM = Ti, V, Cr, Mn, Fe, Co, Ni, Cu, Zn, Pd, Ag, Pt, Au	DFT	HCHO	[0.83, 2.01]
Cu+1N-GNR [150]	DFT	H_2_	−0.020
Cu+2N-GNR [150]	DFT	H_2_	−0.200
Cu+3N-GNR [150]	DFT	H_2_	−0.780
TiO_2_-GO [149]	Experimental	H_2_	Not reported
Ni-G [48]	DFT	H_2_S CH_4_	−0.699 −0.099
TM-BG [53] TM = V, Cr, Mn, Fe, Co, Ni	DFT	H_2_S	[−0.58, −0.18]
Pt_13_-G [153]	DFT	CO_2_ NO_2_ SO_2_	[−4.217, −2.422] [−3.767, −2.586] [−3.260, −2.238]
Pt_13_-DG [153]	DFT	CO_2_ NO_2_ SO_2_	[−3.201, −0.916] [−3.345, −2.309] [−2.978, −2.065]
Mn-G [54]	DFT	CH_4_ C_2_H_2_ CO	−0.073 −2.424 −1.954
Ni-G [46]	DFT	NO_2_ H_2_S	[−2.631, −2.395] [−1.846, −1.811]
Pd-G [46]	DFT	NO_2_ H_2_S	[−1.586, −1.294] [−1.228, −1.224]
Pt-G [46]	DFT	NO_2_ H_2_S	[−2.003, −1.804] [−2.034, −1.858]
Pd-G [55]	DFT	CO NO	[−1.227, −0.909] [−3.916, −1.308]
Ag-G [56]	DFT	NO NO_2_	[−6.9262, −6.9101] [−7.8293, −7.7806]
Pt-G [56]	DFT	NO NO_2_	[−6.2225, −6.1646] [−7.3758, −7.3723]
Au-G [56]	DFT	NO NO_2_	[−8.4730, −8.3567] [−9.3391, −9.3209]
TM-G [57] TM = Ti, Mn, Fe, Co, Ni, Ag	DFT	AsH_3_ CO	[−0.95, −1.45] [−1.00, 2.02]
Ni-G [58]	DFT	H_2_S	−0.97
Cu-G [58]	DFT	H_2_S	−1.15
Zn-G [58]	DFT	H_2_S	−1.16
TM-G [151] TM = Fe, Ni, Co, Cu	DFT	CO_2_ NO NO_2_ SO_2_	[−0.89, −1.19] [−0.68, −1.23] [−2.06, −2.57] [−0.89, −1.47]
Fe- AGNR [152]	DFT	CO CO_2_ NO NO_2_	−2.4 −1.3 −3.1 −3.0
Zr-G [59] Mo-G [59] Ti-G [59] Mn-G [59] Fe-G [59] Co-G [59]	DFT	COCl_2_	−0.894 −0.960 −1.065 −1.677 −1.378 −0.828
TM-GNF [60] TM = Sc, Ti, V, Cr, Mn, Fe, Co, N, Cu, Zn	DFT	CO CO_2_	[−8.13, −37.56] [ −5.05, −16.11]
MN_4_-G [155] M = Sc, Ti, V, Cr, Mn, Co, Ni, Cu, Zn	DFT	CO_2_	[−0.0032, −0.0125]

**Table 9:** Summary of adsorption energies E_ads_ (in eV) for different TM-doped fullerene systems and adsorbates. The type of study is also specified.

System	Type of Study	Adsorbate	E_ads_
Ti-C_60_ [156]	DFT	H_2_	−0.14
TM-C_60_ [79] TM = Cr, Mn, Fe, Co, Ni, Cu; Zn	DFT	CO NO	[−2.94, −1.03] [−6.52, −1.95]
Cr-C_70_ [158]	TD-DFT	NO_2_ SO_2_	[−1.55, −0.605] [−1.178, −0.025]
Co-C_70_ [158]	TD-DFT	NO_2_ SO_2_	[−1.919, −0.806] [−0.627, −0.0047]
M_x_-C_60_ [159] M = Mn, Co, Ni, Cu (x = 2–5)	DFT	CH_3_OH HCOOH CH_3_CH_2_OH O_2_ CO SO_2_	[−0.26, −0.17] [−0.19, −0.08] [−0.27, −0.16] [−0.19, −0.10] [−0.35, −0.12] [−0.22, −0.09]
Co_n_-C_60_ [157] (n = 1–8)	DFT	H_2_ 2H	[1.31, 0.60] [1.78, 1.08]
TM-C_60_ [67] TM = Cu, Zn, Ni	DFT	N_2_O CO	[−2.30, −1.41] [−3.5, −1.56]

**Table 10:** Summary of adsorption energies E_ads_ (in eV) for different TM-doped graphdiyne systems and adsorbates. The type of study is also specified.

System	Type of Study	Adsorbate	E_ads_
TM-GDY [98] TM = Li, Na, K, Rb, Cs	DFT	CO_2_	[−0.54, −0.21]
Cu-GDY [100] Cu-B-GDY [100] Cu-N-GDY [100]	DFT	CO_2_	[−0.5, −0.4] [−0.45, −0.3] [−0.5, −0.31]
Pt-GDY [103]	DFT	O_2_ CO	−1.14 −1.56
Ni-GDY [168]	DFT	O_2_ CO O_2_ + CO O CO_2_	−0.69 −1.68 −1.09 −3.21 −0.08
Cu-GDY [168]	DFT	O_2_ CO O_2_ + CO O CO_2_	−0.79 −1.25 −1.43 −3.21 −0.37
Sc-GDY [165] Ti-GDY [165]	DFT	HCHO	−2.59 −2.24
TM-GDY [161] TM = Ti, Sc, Li, Na, K, Ca	DFT (GGA) DFT (vdW-DF) DFT (DFT-D3)	8H_2_	[−0.197, −0.10] [−0.77, −0.194] [−0. 345, −0.173]
Mo-GDY [163]	DFT	N_2_	[−1.35, −0.93]
TM-GDY [82] TM = Sc, Ti, V, Cr, Mn, Fe, Co, Ni, Cu, Y, Zr, Rh, Pd, Ag, La, Hf, Pt	DFT	N_2_	[−1.8, +0.12]
TM-GDY [160] TM = Cr, Mn, Fe, Co	DFT-D3	O_2_ CO	[−2.52, −1.21] [−1.57, −1.25]
TM-1N-GDY [160] TM = Cr, Mn, Fe, Co	DFT-D3	O_2_ CO	[−2.56, −1.07] [−1.78, −1.21]
TM-2N-GDY [160] TM = Cr, Mn, Fe, Co	DFT-D3	O_2_ CO	[−2.31, −1.17] [−1.94, −1.39]
Ru-GDY [162]	Experimental	H_2_O	Not reported
Fe-GDY [167]	DFT-D3/AIMD	CO_2_	Not reported

**Table 11:** Summary of adsorption energies E_ads_ (in eV) for different TM-metallic doped hybrid systems and adsorbates. The type of study is also specified.

System	Type of Study	Adsorbate	E_ads_
Ti-C_30_ [171]	DFT	CO CO_2_	[−0.897, −1.673] [−1.605, −1.247]
Ti-G-Semifullerene [172]	DFT	H_2_	−1.41
Ni-, Fe-CNOs [170]	Experimental	H_2_	Not reported

## Data Availability

Not applicable.
